# The relationship between perceived motivational climate and psychological well-being among adolescent handball players

**DOI:** 10.3389/fspor.2026.1827113

**Published:** 2026-06-18

**Authors:** Zrinka Greblo Jurakić, Lidija Bojić-Ćaćić, Višnja Ljubičić

**Affiliations:** 1 Department of Psychology, Faculty of Croatian Studies, University of Zagreb, Zagreb, Croatia; 2 Croatian Handball Federation, Zagreb, Croatia; 3 Croatian Ombudswoman for Gender Equality, Zagreb, Croatia

**Keywords:** handball, motivational climate, self-esteem, anxiety, depression, stress

## Abstract

**Introduction:**

Sport participation can both enhance and undermine adolescents’ well-being. Therefore, the aim of this study was to examine associations of perceived task- and ego-involving motivational climates with anxiety, depressive, and stress symptoms and global self-esteem among youth athletes, and to test whether perceived motivational climate varied by athlete and coach gender.

**Methods:**

A total of 522 handball players (aged 14–17 years) participated in the study. Participants completed the Perceived Motivational Climate in Sport Questionnaire-2, Depression Anxiety Stress Scales (DASS-21), and the Rosenberg Self-Esteem Scale.

**Results:**

Overall, handball players perceived their teams as more task- than ego-involving, although male athletes reported lower task-involving climate than female athletes. Perceived task-involving climate was associated with lower anxiety, depressive, and stress symptoms and higher self-esteem, whereas ego-involving climate showed the opposite pattern. Together, motivational climate variables accounted for a statistically significant proportion of variance in anxiety, depressive, and stress symptoms, as well as self-esteem. In regression analyses, higher task-involving climate was associated with higher self-esteem, whereas higher ego-involving climate was associated with lower self-esteem and showed stronger associations with anxiety, depressive, and stress symptoms. Among female athletes, those coached by men perceived lower task-involving and higher ego-involving climates than those coached by women.

**Conclusion:**

Fostering a task-involving climate while minimising ego-involving cues may be relevant for supporting youth athletes’ psychological well-being. The findings highlight the importance of coach education that addresses the psychological risks of an ego-involving motivational climate and promotes coaching practices that foster supportive, task-involving environments for youth handball players.

## 1 Introduction

A growing body of research suggests that sport participation during childhood and adolescence is associated with many psychosocial benefits that extend beyond physical activity alone (1, 2). Consistent with the view that sport involvement may play a protective role for youth mental health, a systematic review and meta-analysis conducted by Panza and colleagues (2) showed that sport participation during adolescence was associated with lower levels of anxiety and depressive symptoms. These results are especially important in the context of increased prevalence of mental health difficulties among young people (3, 4). Taken together, empirical findings highlight the need to better understand the potential of sport participation as a means of reducing the risk of psychological difficulties and supporting children's and adolescents’ psychosocial adjustment.

Results from several studies suggest that, with respect to psychosocial well-being, participation in team sports might be especially beneficial (5–9). The observed pattern may be attributed to the distinct social aspects of team sports, which can foster peer support and collaboration in the pursuit of shared goals (1, 7). Underscoring the importance of interpersonal processes in shaping positive mental health outcomes is consistent with evidence that the benefits of sport participation do not occur automatically, but rather depend on specific characteristics of social environment (10). According to Fraser-Thomas and colleagues (11) beneficial effects are more likely to occur in sport environments that actively promote developmental, relational, and educational values through positive social norms, supportive relationships, and skill-building opportunities. On the other hand, sport environments characterised by excessive performance pressure and unsupportive, overly competitive, or violent interpersonal relationships might significantly undermine athletes' psychosocial well-being (12–15).

Theoretical models (16, 17) and empirical findings (18) indicate that social variables affect athletes' perceptions, interpretations, emotions, and behaviours in achievement settings. Achievement Goal Theory (17) provides a robust framework for understanding how specific norms that define the concept of success might shape psychological outcomes associated with sport participation. Ames (16) specified two forms of motivational climate: mastery or task-involving climate and performance or ego-involving climate, which are related to distinct affective and self-evaluative processes. A task-involving motivational climate emphasises the importance of learning and personal improvement, promotes self-referenced standards of success, rewards effort and fosters cooperation between team members. By contrast, an ego-involving motivational climate is focused on results and outperforming others. It relies on norm-referenced criteria of success, social comparison, and punishment for mistakes (19). Adoption of distinct normative criteria for success may lead to different affective and self-evaluative processes among athletes (10, 16, 18). For example, when competence is framed in self-referenced or task-referenced terms, as promoted within a task-involving climate, athletes are more likely to perceive mistakes as an inevitable part of the learning process and as cues signalling the need to invest more effort or adjust their learning strategies. On the other hand, when competence is framed in norm-referenced terms, as in an ego-involving climate, mistakes might be perceived as indicators of low ability and, consequently, trigger threat appraisal and stress-related affect. Consistent with this reasoning, empirical findings showed that coach ego-involving motivational climate was associated with higher levels of trait anxiety among youth athletes (20). In addition, lower levels of self-esteem were found among athletes with low perceived ability who perceived their team climate as highly ego-involving, whereas perceiving a highly task-involving climate was associated with higher self-esteem regardless of perceived ability (10).

Drawing on Achievement Goal Theory (17), the present study conceptualises perceived motivational climate as a central feature of the adolescent sport environment that may shape how youth athletes understand competence, effort, mistakes, success, and failure. From this perspective, task- and ego-involving climates are not seen merely as different motivational settings, but as qualitatively different psychological contexts that communicate how effort is valued, how competence is judged, and how success and failure are defined in sport (18, 19). These differences may be especially important during adolescence, when self-concept, peer evaluation, and sensitivity to interpersonal feedback become increasingly salient (21, 22). Accordingly, motivational climate and other relational features of the sport environment may be relevant not only to athletes' sport-specific experiences, but also to broader psychosocial functioning and well-being-related outcomes (23–26). Motivational climate may be particularly salient for adolescents participating in team sports, where athletes' experiences are embedded in perceived team practices and the shared motivational atmosphere of the group. Given the highly interactive and team-based nature of the game, youth handball provides a relevant setting for examining how organised sport participation is associated with athletes' mental health and psychosocial functioning. From a practical perspective, findings from this line of research may help inform coach education and youth sport programmes by identifying ways to create motivational climates that support both athletic development and psychosocial well-being.

Although motivational climate has received considerable research attention in sport and physical activity contexts, earlier empirical studies focused primarily on sport-related motivational, affective, and self-evaluative outcomes (18). Consistent with reviews highlighting the need to identify factors that may contribute to both beneficial and potentially harmful outcomes of youth sport participation (2, 25, 27, 28), more recent studies have extended motivational climate research to athletes' well-being (26, 29–33). However, fewer studies have examined whether task- and ego-involving climates are associated with broader indicators of mental health and psychosocial functioning, despite the relevance of these outcomes for understanding when sport environments may support or undermine adolescents' psychosocial adjustment. The present study addresses this gap by examining associations between task- and ego-involving motivational climates and anxiety, depressive, and stress symptoms, as well as global self-esteem, among adolescent handball players. Based on current theoretical and empirical evidence, we hypothesised that perceived task-involving motivational climate would be negatively associated with anxiety, depressive, and stress symptoms and positively associated with global self-esteem. This hypothesis was grounded in the view that a task-involving climate emphasises effort, improvement, cooperation, and constructive responses to mistakes, thereby creating conditions that may support more adaptive self-evaluative and affective experiences. In contrast, we hypothesized that an ego-involving motivational climate would show the opposite pattern of associations, because its emphasis on normative comparison, unequal recognition, rivalry, and punishment for mistakes may increase evaluative pressure and make mistakes feel more threatening to athletes' sense of competence and self-worth. In addition, given evidence that demographic and social-contextual factors may shape youth sport experiences (2, 25, 27), we examined whether perceived motivational climate differed by athlete gender and, among female athletes, by coach gender.

## 2 Materials and methods

### 2.1 Participants

A total of 522 handball players (289 male, 233 female) participated in the study (age range: 14–17; *M* = 14.75; *SD* = 0.96). On average, participants started playing handball when they were 8 years old (*M* = 8.55; *SD* = 1.97) and, at the time of the study, had on average 6 years of playing experience (*M* = 6.30; *SD* = 2.12). All athletes participated in competitions at the national (80%) or international level (20%). Most of the male athletes (91.2%) were coached by men, whereas female athletes were evenly split between male (50.4%) and female (49.6%) coaches.

### 2.2 Procedure

The findings reported in the present study are part of a larger research project examining psychosocial factors associated with youth handball participation. The study was conducted during the national finals competition. Two weeks before data collection, in collaboration with the Croatian Handball Federation, parents/legal guardians of young athletes were informed about the aims of the study and the data collection procedure. Participation in the study was anonymous and voluntary. Before completing the questionnaires, all participants provided written informed consent for study participation. Young athletes filled out the paper-and-pencil questionnaires in rooms prepared to ensure adequate conditions for standardized group testing. The entire survey required approximately 15 min to complete. Following questionnaire completion, the lead researcher was available to answer questions related to the research topic. The study was approved by the Ethics Committee of the Croatian Psychological Chamber (Reference No. 251-375/01-04-19-2) and was conducted in accordance with the Code of Ethics for Research with Children (34).

### 2.3 Measures

The Perceived Motivational Climate in Sport Questionnaire-2 (PMCSQ-2) (19) was used to assess athletes' perceptions of the motivational climate in their team. The PMCSQ-2 is a 33-item measure comprising two higher-order dimensions: task-involving climate and ego-involving climate, each consisting of three subscales. The task-involving dimension includes the Cooperative Learning, Effort/Improvement, and Important Role subscales, which capture the extent to which team members support one another's learning, value personal progress and effort, and perceive that all players make meaningful contributions to the team (e.g., “*On this team, each player contributes in some important way*”.). The ego-involving dimension includes the Unequal Recognition, Intra-Team Member Rivalry, and Punishment for Mistakes subscales, reflecting aspects of a team environment in which greater recognition is given to the best-performing athletes, teammates are encouraged to compare and compete with one another, and mistakes are followed by negative reactions (e.g., “*On this team, players are punished when they make a mistake*”.). Participants indicated their responses on a 5-point scale ranging from 1 (*strongly disagree*) to 5 (*strongly agree*). The PMCSQ-2 was translated from English into Croatian using a translation-back-translation procedure to ensure linguistic and conceptual equivalence (35). In the present study, Cronbach's alpha coefficients for the higher-order Task- and Ego-involving climate scores were .88 and .90, respectively. Internal consistency coefficients for the subscales were .72 for Cooperative Learning, .80 for Effort/Improvement,.64 for Important Role, .77 for Punishment for Mistakes, .88 for Unequal Recognition, and .38 for Intra-Team Member Rivalry. Although most subscales demonstrated acceptable internal consistency, two coefficients required additional consideration. The Important Role subscale was retained in the analyses because it captures a theoretically relevant aspect of task-involving climate, although findings involving this subscale should be interpreted cautiously given its marginal reliability. Consistent with previous psychometric evidence indicating weaker reliability for this dimension (19, 36) the Intra-Team Member Rivalry subscale showed poor internal consistency in the present sample and was therefore excluded from subscale-level analyses. However, in line with the questionnaire's higher-order structure (19), its items were retained in the Ego-involving climate total score. Accordingly, motivational climates were operationalised through the higher-order Task- and Ego-involving scores and the subscales: Important Role, Cooperative Learning, Effort/Improvement (Task-involving subscales) and Punishment for Mistakes and Unequal Recognition (Ego-involving subscales).

The short version of the Depression Anxiety Stress Scales (DASS-21) (37) was used to measure depressive, anxiety, and stress symptoms experienced by athletes over the past week. The scale consists of 21 items rated on a 4-point scale ranging from 0 (*did not apply to me at all*) to 3 (*applied to me very much, most of the time*). Representative items include: “*I couldn't seem to experience any positive feeling at all*”. (Depression subscale), “*I felt scared without any good reason*” (Anxiety subscale), and “*I tended to over-react to situations*”. (Stress subscale). The DASS-21 has previously been used in Croatian research to assess depressive, anxiety, and stress symptoms among adolescents and young adults (38–40). In the present study, Cronbach's alpha coefficients were .85 for the depressive symptoms, .81 for the anxiety symptoms, and. 82 for the stress symptoms.

The Rosenberg Self-Esteem Scale (41) was used to assess global self-esteem among youth handball players. The scale consists of 10 items capturing overall positive and negative evaluations of the self (e.g., “*On the whole, I am satisfied with myself*”.). For each item, participants indicated their level of agreement on a 4-point scale (1—*strongly disagree*; 4—*strongly agree*), with higher scores indicating higher global self-esteem after reverse scoring of negatively worded items. Previous studies using the Rosenberg Self-Esteem Scale in Croatian adolescent samples have reported good internal consistency (42, 43). In the present study, Cronbach's alpha coefficients were .85 for the depressive symptoms, .81 for the anxiety symptoms, and .82 for the stress symptoms.

## 3 Results

Compared with male athletes, female athletes reported higher levels of anxiety and stress symptoms and lower levels of global self-esteem. No gender differences were observed in depressive symptoms (Table 1).

**Table 1:** Gender differences in mental health indicators (anxiety, depressive and stress symptoms), global self-esteem, and perceived task and ego-involving motivational climates (MC).

		Male handball players			Female handball players		*t-test*
Variables	*n*	*M*	*SD*	*n*	*M*	*SD*	
1. Anxiety symptoms	265	5.25	6.74	224	7.66	7.90	−3.601***
2. Depressive symptoms	265	5.84	7.42	217	6.63	8.40	−1.076
3. Stress symptoms	270	8.53	8.14	223	10.60	8.79	−2.707**
4. Self-esteem	259	3.12	0.48	216	2.97	0.56	3.082**
5. Task MC total score	220	4.17	0.591	192	4.33	0.534	−2.877**
6. Important Role	249	4.01	0.705	215	4.12	0.666	−1.617
7. Cooperative Learning	261	4.06	0.802	220	4.26	0.698	−2.934**
8. Effort/Improvement	236	4.26	0.667	212	4.44	0.548	−3.197***
9. Ego MC total score	215	2.88	0.870	201	2.77	0.807	1.358
10. Punishment for Mistakes	243	2.96	0.917	222	2.85	0.827	1.307
11. Unequal Recognition	244	2.68	1.016	211	2.69	1.065	−0.119

** *p* ≤ .01; ***p ≤ .001.

Analyses of differences in the perception of task and ego-involving climates revealed that male athletes scored significantly lower on the task-involving climate total scale, as well as on the Cooperative Learning and Effort/Improvement subscales. Male and female athletes did not differ significantly in their perceptions of ego-involving motivational climate (Table 1).

Paired-samples *t*-tests were then conducted separately for male and female athletes to compare total scores for perceived task-involving and ego-involving motivational climates. Male athletes reported higher task-involving climate scores (*M* = 4.19, *SD* = 0.58) than ego-involving climate scores (*M* = 2.89, *SD* = 0.88), *t*(185) = 15.07, *p* < .001. The same pattern was found among female athletes, with higher task-involving (*M* = 4.33, *SD* = 0.54) than ego-involving scores (*M* = 2.76, *SD* = 0.81), *t*(170) = 18.55, *p* < .001.

As shown in Table 2, anxiety, depressive, and stress symptoms were strongly and positively intercorrelated, whereas self-esteem showed a moderate negative correlation with each symptom dimension. Task- and ego involving motivational climate total scores were also moderately negatively correlated.

**Table 2:** Partial correlations between study variables, adjusted for gender.

Variables	1.	2.	3.	4.	5.	6.	7.	8	9.	10.	11.
1. Anxiety symptoms	–										
2. Depressive symptoms	.62***	–									
3. Stress symptoms	.72***	.67***	–								
4. Self-esteem	−.33***	−.49***	−.34***	–-							
5. Task Climate total score	−.16**	−.18**	−.14*	.26***	–						
6. Important Role	−.14*	−.13*	−.12*	.20***	.85***	–					
7. Cooperative Learning	−.15**	−.17**	−.17**	.25***	.84***	.60***	–				
8. Effort/ Improvement	−.14*	−.17**	−.09	.24***	.92***	.65***	.67***	–			
9. Ego Climate total score	.37***	.32***	.32***	−.30***	−.33***	−.32***	−.34***	−.24***	–		
10. Punishment for Mistakes	.33***	.31***	.30***	−.38***	−.31***	−.27***	−.34***	−.22***	.88***	–	
11. Unequal Recognition	.35***	.30***	.31***	−.25***	−.39***	−.38***	−.37***	−.29***	.95***	.76***	–

* *p* ≤ .05; **p ≤ .01; ***p ≤ .001.

The total score on the Task-involving motivational climate scale showed weak negative correlations with anxiety, depressive and stress symptoms, and a positive correlation with global self-esteem (Table 2). At the subscale level, Important Role and Cooperative Learning showed the same pattern, while Effort/Improvement was negatively correlated with anxiety and depressive symptoms and positively correlated with self-esteem. In contrast, the Ego-involving motivational climate total score and the included ego-involving subscales showed moderate positive correlations with anxiety, depressive, and stress symptoms, and negative correlations with self-esteem.

Eight separate multiple regression analyses were conducted to examine whether task- and ego-involving motivational climate total scores explained a statistically significant proportion of variance in self-reported anxiety, depressive and stress symptoms, as well as global self-esteem (Table 3).

**Table 3:** Regression analyses examining associations between perceived motivational climate (MC), mental health indicators, and global self-esteem.

	Anxiety symptoms			Depressive symptoms			Stress symptoms			Self-esteem		
Variables	*β*	*R^2^_adj._*	*F*	*β*	*R^2^_adj._*	*F*	*β*	*R^2^_adj._*	*F*	*β*	*R^2^_adj._*	*F*
Male athletes												
Task-involving MC	−.16*	.15	16.30***	−.18*	.17	19.12***	−.06	.14	15.61***	.24***	.14	15.69***
Ego-involving MC	.32***			.34***			.36***			−.25***		
Female athletes												
Task-involving MC	.02	.14	14.40***	−.03	.06	6.55**	−.03	.05	5.55**	.18*	.07	7.34***
Ego-involving MC	.40***			.27***			.24**			−.18*		

* *p* ≤ .05; **p ≤ .01; ***p ≤ .001.

As shown in Table 3, all regression models examining perceived motivational climate in relation to psychological distress symptoms and self-esteem were statistically significant. The task- and ego-involving motivational climate accounted for 15% and 14% of the variance of anxiety symptoms; 17% and 6% of the variance of depressive symptoms; 14% and 5% of the variance of stress symptoms; and 14% and 7% of the variance of global self-esteem, among male and female athletes, respectively. Perceived ego-involving motivational climate was positively associated with anxiety, depressive, and stress symptoms in both male and female athletes. In addition, perceived task-involving climate was negatively associated with anxiety and depressive symptoms among male athletes. Finally, higher self-esteem was associated with perceiving a more task-involving and less ego-involving motivational climate among both male and female handball players.

Finally, we examined whether female athletes' perceptions of team motivational climate differed as a function of coach gender (Table 4). The analysis was restricted to female athletes because coach gender was nearly evenly distributed in the female subsample (50.4% male coaches and 49.6% female coaches). In contrast, only 8.8% of male athletes (*n* = 25) reported being coached by a female coach, so the male subsample was not suitable for this kind of comparative analysis.

**Table 4:** Differences in female athletes’ perceptions of task- and ego-involving motivational climate by coach gender.

Perceived motivational climate		Coach gender					
		Male coach			Female coach		*t-test*
Variables	*n*	*M*	*SD*	*n*	*M*	*SD*	
1. Task Climate total score	95	4.23	0.577	89	4.41	0.477	−2.357*
2. Important Role	105	4.03	0.681	102	4.18	0.637	−1.615
3. Cooperative Learning	108	4.16	0.732	103	4.34	0.662	−1.920
4. Effort/Improvement	103	4.37	0.598	100	4.51	0.502	−1.880
5. Ego Climate total score	102	2.88	0.832	92	2.63	0.787	2.195*
6. Punishment for mistakes	109	3.02	0.861	104	2.67	0.785	3.108*
7. Unequal recognition	107	2.81	1.097	97	2.54	1.047	1.748

* *p* ≤ .05.

Compared with those coached by women, female athletes coached by men reported significantly lower task-involving climate total scores and significantly higher ego-involving climate total scores. In addition, female athletes coached by male coaches reported higher levels of perceived punishment for mistakes (Table 4).

## 4 Discussion

Empirical evidence indicates that adolescence is a sensitive period for the onset of social-emotional mental health disorders (21), highlighting the importance of identifying factors that may enhance or undermine young athletes' psychological well-being. In the present study, young female athletes reported higher anxiety and stress symptoms and lower global self-esteem. This pattern aligns with findings from outside the sport indicating that, relative to boys, girls report higher anxiety (44, 45) and stress levels (46–48), as well as lower levels of self-esteem (49). Similar results have also been found in sport setting, with young female athletes reporting higher levels of precompetitive anxiety (50) and stress symptoms (51), alongside lower self-esteem (52, 53). Although developmental and biological processes may play a role (54), research suggests that psychosocial factors such as differential treatment, societal pressure, gender stereotypes and expectations might substantially contribute to observed differences (46, 47, 49, 54, 55). No gender differences were observed in depressive symptoms suggesting that gender differences in distress-related symptoms among adolescent athletes might be more pronounced for anxiety and stress. According to systematic review and meta-analysis of mental health symptoms among elite athletes (56), female athletes show higher risk of moderate to severe depression, whereas male and females show similar risk of mild depression. Thus, the absence of gender differences in depressive symptoms in the present study may reflect a restricted range of depressive symptoms among young handball players (e.g., relative few moderate-severe cases). Alternatively, it is possible that gender gap in depression is not yet fully established in early to mid-adolescence. Taken together, the findings underscore the importance of monitoring mental health in youth sport for all athletes, while also recognizing that female athletes may warrant particular attention regarding anxiety/stress and self-evaluative functioning. Correlation analyses indicated that athletes' distress symptoms were moderately and inversely associated with global self-esteem. This finding is consistent with broader evidence linking higher self-esteem with fewer psychological problems and better well-being in youth (57).

Male and female handball athletes described their team climate more in task-involving than ego-involving terms implying that, in their teams, more emphasis is put on effort, personal improvement and support then on normative comparison, intrateam competition and punishment for mistakes. These results are consistent with previous findings showing that adolescent athletes perceived their team climate as predominantly task-involving (10). Similarly, in a study that examined the effects of coach created motivational climate, young handball players also reported higher levels of a task-involving and lower levels of an ego-involving climate (58). In the present sample, females reported higher perceived task-involving climate, especially in terms of cooperative learning and the emphasis on effort and personal improvement. Previous research have also found that young female athletes perceive motivational climate as more task-involving than their male counterparts (20, 59), suggesting that in female teams more emphasis is put on teammate support and cooperation in acquiring new skills, while success is more often framed in self-referenced terms. Given that the perception of a task-involving motivational climate is associated with a variety of positive psychosocial outcomes (18), these findings underscore the importance of strengthening coaching practices that foster task-involving climate in male teams. Male and female athletes did not differ with respect to their perception of ego-involving motivational climate. According to Achievement Goal Theory (60, 61), adolescents' increasing cognitive maturity may heighten sensitivity to performance-evaluative cues that are often embedded in competitive sport. Consequently, such shared exposure may contribute to similar perceptions of ego-involving cues among male and female athletes.

Perceived task-involving climate was associated with a more favourable well-being profile among youth handball players (lower anxiety, depressive, and stress symptoms and higher global self-esteem), with small negative correlations obtained for distress symptoms and small-to-moderate positive correlations obtained for self-esteem. At the subscale level, the analyses showed that young handball players who perceived their team climate as one that values each player's role and fosters peer-supported learning reported less psychological distress and higher self-esteem. Moreover, perceiving a climate that recognises contributions across ability levels, and emphasises skill development and personal improvement was associated with lower anxiety and depressive symptoms and higher global self-esteem. The results build on previous studies showing that task-involving motivational climate is associated with more adaptive psychosocial outcomes, including adaptive perfectionism, lower levels of burnout and performance anxiety, higher resilience, and greater self-confidence (62–65). On the other hand, perceived ego-involving motivational climate showed moderate positive associations with anxiety, depressive and stress symptoms and negative associations with self-esteem. Once again, the subscales mirrored the pattern: athletes who were part of the team in which players were punished for mistakes, coaches responded with anger after errors, and some players were favoured over others, reported lower self-esteem and higher levels of anxiety, depressive, and stress symptoms. Earlier empirical findings also indicate that emphasising differences in abilities, encouraging social comparison, and punishing athletes for mistakes can have negative effects on athletes' well-being (66). Consistent with mentioned, in previous studies ego-involving climate was associated with maladaptive perfectionistic tendencies, burnout, and anxiety (20, 64, 67).

Perceived task- and ego-involving climates explained significant variance in distress symptoms and self-esteem among youth handball players. In the male subsample, both task- and ego-involving climates were significantly associated with anxiety and depressive symptoms in the regression models, with task-involving climate showing negative associations and ego-involving climate showing positive associations. In the female subsample, anxiety and depressive symptoms were significantly associated only with ego-involving climate. Across both subsamples, only ego-involving climate was significantly associated with stress symptoms. Self-esteem was positively associated with task-involving climate and negatively associated with ego-involving climate in both groups. Overall, ego-involving climate emerged as the more robust correlate of distress, whereas task-involving climate showed a small inverse association with anxiety and depressive symptoms among males, suggesting its possible protective role. These findings align with empirical studies that highlight the importance of supportive and cooperative behaviours for athletes' well-being and adjustment in sport environment (32, 62, 64), as well as with findings showing that ego-involving motivational climate might undermine athletes' psychological well-being (10, 18, 23). Perceived task- and ego-involving motivational climates explained a comparable proportion of variance in anxiety symptoms across genders. In contrast, the models accounted for more variance in depressive and stress symptoms, as well as self-esteem, among males than females. Although findings are not consistent across studies, evidence from dual-career literature suggests that, compared with their female counterparts, male student-athletes report stronger athletic identities (68). Thus, one plausible explanation for our finding is that perceived motivational climate may contribute more to male athletes' well-being when sport is more closely tied to their identity, whereas females' psychosocial adjustment may reflect a broader set of influences beyond the sport context. However, since athletic identity was not measured in the present study, this interpretation remains speculative and should be examined in future research.

Finally, compared with those coached by women, female athletes coached by men perceived a lower task-involving and higher ego-involving team climate. At the subscale level, they also reported higher perceived punishment for mistakes. These findings are broadly consistent with research suggesting that, on average, male and female coaches may differ in interpersonal coaching styles. For example, in a qualitative study, elite female soccer players reported that male coaches tend to have a rougher, more “masculine” communication style, whereas female coaches were perceived as more understanding and caring (69). In a more recent study conducted on a sample of youth and high school coaches (70), women coaches scored significantly higher on autonomy-supportive behaviours, which are conceptually consistent with task-oriented cues and associated with more adaptive psychosocial outcomes among youth athletes (71).

From a practical perspective, the findings suggest that coach education in youth handball should emphasise process-focused feedback, framing mistakes as an integral part of learning, valuing each player's contribution, recognising effort and improvement across ability levels, and encouraging teammate support and cooperation. On the other hand, coaches should be encouraged to minimise ego-involving cues, such as unequal recognition, frequent normative comparison, excessive competitiveness among team members, and punitive responses to mistakes. These implications are consistent with recent youth sport and athlete mental health literature showing that coach- and peer-created motivational climates, as well as broader interpersonal features of the sport environment are relevant for sport continuation, psychosocial development and youth athletes' mental health related outcomes (15, 32, 72).

Research findings should be interpreted in light of several limitations. First, because the study was conducted during the national finals competition, the sample consisted mostly of higher-performing youth athletes. To obtain a more comprehensive understanding of motivational climate correlates, future studies should include athletes competing at different levels. Second, the cross-sectional design precludes causal inference and does not allow conclusions about temporal ordering. Longitudinal research is therefore needed to test whether perceived task- and ego-involving climates are associated with subsequent changes in well-being indicators over time. Third, all variables were assessed using self-report questionnaires, which may increase the risk of common method bias and shared response variance, particularly when independent and dependent variables are measured using the same response method (73, 74). Therefore, future studies should consider multi-method and multi-informant designs that complement athletes' self-reports with additional data sources, such as observational assessments of motivational climate and coach reports. Finally, although most PMCSQ-2 subscales (19) showed acceptable internal consistency, reliability concerns emerged for two lower-order subscales. The Important Role subscale showed marginal internal consistency; therefore, findings involving this subscale should be interpreted with caution. In addition, due to poor internal consistency, the Intra-Team Member Rivalry subscale was excluded from subscale-level analyses. Consequently, we were unable to examine how perceived competition between team members was associated with mental health indicators and global self-esteem among youth athletes. Future studies should further examine the psychometric performance of PMCSQ-2 subscales in adolescent sport samples and consider whether some lower-order dimensions require refinement, cultural adaptation, or additional validation. Despite these limitations, the findings contribute to the existing body of evidence suggesting that a task-involving motivational climate may be associated with more favourable psychosocial well-being among youth athletes, whereas an ego-involving motivational climate may be associated with a less favourable well-being profile.

## Data Availability

The datasets presented in this article are not readily available because they contain sensitive data. Requests to access the datasets should be directed to Zrinka Greblo Jurakić, zgreblo@fhs.hr.
